# High-Dose Nitroglycerin in Managing Sympathetic Crashing Acute Pulmonary Edema (SCAPE): A Comprehensive Review of Recent Evidence and Clinical Outcomes

**DOI:** 10.7759/cureus.92262

**Published:** 2025-09-14

**Authors:** Mohamed E Abdelhameed, Qais Ababneh, Osman Amir, Mohamed M Mustafa, Osama M Abdulhai

**Affiliations:** 1 Emergency Medicine Department, Al Qassimi Hospital, Sharjah, ARE; 2 Emergency Medicine Department, Jordan University of Science and Technology, Irbid, JOR; 3 Hematology Department, Karary University, Omdurman, SDN; 4 Emergency Department, Sheikh Tahnoon bin Mohammed Medical City, Al Ain, ARE; 5 Emergency Department, Dongola Specialized Hospital, Dongola, SDN

**Keywords:** acute heart failure, hdn, hemodynamic stabilization, high-dose nitroglycerin, icu admission, intubation, mace, scape, sympathetic crashing acute pulmonary edema, vasodilator therapy

## Abstract

This review evaluated recent clinical evidence on the management of sympathetic crashing acute pulmonary edema (SCAPE) with high-dose nitroglycerin (HDN) therapy. Several recent studies, including randomized controlled trials and cohort studies, were analyzed to assess the efficacy and safety of HDN compared with low-dose nitroglycerin (LDN). Across studies, HDN was associated with improved clinical outcomes, including reduced rates of ICU admission, intubation, and acute kidney injury; shorter hospital stays; a lower incidence of major adverse cardiac events; and more rapid achievement of blood pressure targets. Symptom resolution within six hours was consistently reported, and most patients were discharged without complications. Adverse effects, such as hypotension, were infrequent. Collectively, these findings support the use of HDN as a safe and effective intervention for SCAPE, warranting further large-scale randomized trials to confirm these promising results.

## Introduction and background

Sympathetic crashing acute pulmonary edema (SCAPE) represents a critical emergency characterized by abrupt-onset respiratory distress, severe hypertension, and pulmonary edema secondary to acute heart failure exacerbated by excessive sympathetic activation [1,2]. This condition demands immediate intervention to disrupt the self-perpetuating cycle of elevated afterload, fluid redistribution into pulmonary tissues, and compensatory catecholamine surge. Traditional therapeutic approaches emphasizing low-dose nitroglycerin (LDN) infusion (10-100 µg/minute) combined with diuretics have faced scrutiny due to their limited capacity to address the central pathophysiological driver: pathological afterload elevation from systemic vasoconstriction [1-4]. 

Emerging evidence challenges conventional dosing paradigms by demonstrating that high-dose nitroglycerin (HDN) regimens (>100 µg/minute, often administered via intravenous bolus followed by sustained infusion) achieve more rapid hemodynamic stabilization through dual mechanisms of action. While lower doses primarily reduce preload through vasodilation, higher concentrations induce arterial vasodilation, directly targeting the elevated systemic vascular resistance that perpetuates SCAPE's vicious cycle [3-5]. 

The therapeutic shift toward aggressive vasodilation reflects improved understanding of SCAPE's fluid dynamics. Rather than absolute hypervolemia, pulmonary edema arises primarily from acute fluid redistribution caused by afterload-induced ventricular stiffening. HDN addresses this mechanism more effectively than gradual dose escalation, often obviating the need for invasive ventilation and intensive care admission. Recent protocols combining immediate high-dose nitroglycerin boluses with non-invasive ventilation show particular promise, achieving discharge from emergency settings within hours for >95% of patients in observational cohorts [3,4,6]. 

Clinical studies document resolution of respiratory symptoms within three to six hours using cumulative nitroglycerin doses up to 76 mg, with mean initial boluses of 836-872 µg demonstrating both safety and efficacy. This contrasts with historical concerns about hypotension risk, as multiple prospective trials report no significant blood pressure complications when using protocolized high-dose administration [4,6,7]. 

Many clinicians continue to favor the traditional use of LDN in the management of patients with SCAPE, primarily due to concerns regarding the adverse effects associated with high-dose nitroglycerin (HDN). This review aims to synthesize the most recent evidence and critically evaluate the efficacy and safety of HDN compared to LDN in the treatment of SCAPE.

## Review

Methods 

Searching Strategy

A comprehensive search was conducted in PubMed, Europe PMC, and ScienceDirect up to June 2024. The search strategy used the minimal set of keywords - (high dose nitroglycerin) AND (low dose nitroglycerin) AND (pulmonary edema) - to broaden the scope and capture the maximum number of relevant articles. When necessary, additional sources were identified by reviewing the reference lists of selected studies and pertinent review articles. Search terms were adjusted as needed to suit the specific requirements of each database.

Inclusion and Exclusion Criteria

This article review incorporated randomized controlled trials (RCTs), prospective and retrospective observational studies, which examined specific parameters of HDN administration in SCAPE. Studies were selected if they directly compared HDN with LDN in terms of efficacy, safety, and clinical outcomes such as symptom relief, need for mechanical ventilation (MV), hospital length of stay, and major adverse cardiovascular events (MACE) at follow-up.

Inclusion criteria focused on SCAPE patients aged ≥18 years with acute dyspnea (symptom onset <6 hours), systolic blood pressure (SBP) ≥160 mmHg and/or diastolic blood pressure (DBP) ≥100 mmHg (or mean arterial pressure ≥120 mmHg), respiratory rate ≥30 breaths per minute, oxygen saturation (SaO₂) <90%, and signs of sympathetic activation (e.g., tachycardia, diaphoresis) at presentation. Studies were required to provide clear definitions of HDN (exceeding study-specific thresholds) compared to low-dose regimens. Additionally, eligible studies had to report risk estimates with 95% confidence intervals (CI) or include sufficient data to calculate effect sizes for outcomes.

Studies were excluded if they involved patients with non-cardiogenic pulmonary edema; included participants requiring emergent intubation or cardiopulmonary resuscitation; enrolled individuals with nitroglycerin contraindications (hypersensitivity reactions, hypertrophic obstructive cardiomyopathy, moderate-to-severe aortic stenosis, or recent PDE5 inhibitor use (sildenafil ≤24 hours or tadalafil ≤48 hours)); involved pregnant patients; or focused on acute coronary syndrome. Additional exclusions applied to studies lacking essential outcome data, along with non-human research, secondary literature (reviews, editorials), conference materials, and case reports/series.

Measured Outcomes

The main outcomes of this review included the need for MV, the rate of symptom resolution within six hours, the duration of hospital stay, and the risk of MACE. MV is a vital life-support technique used in acute or emergency situations, especially for patients with compromised airways, impaired ventilation, or hypoxemic respiratory failure. This method delivers positive pressure breaths and depends on the compliance and resistance of the airway system. Symptom resolution was defined as meeting at least two of the following three criteria: a reduction in respiratory rate by at least 25% from baseline or a respiratory rate of 24 breaths per minute or less; a SBP of 160 mmHg or lower, DBP of 100 mmHg or lower, or mean arterial pressure of 120 mmHg or lower; and improvement in hypoxia, demonstrated by subjective improvement and oxygen saturation of at least 90% on room air or 95% with supplemental oxygen. MACE was defined as a composite of cardiovascular death, myocardial infarction, ischemic stroke, and rehospitalization for heart failure. For safety, hypotension was defined as a SBP below 90 mmHg, which would prompt immediate discontinuation of nitroglycerin infusion and appropriate medical management.

Results

From 33 selected studies, five articles were considered to be included in this review, published between 2021 and 2024. During the screening, 28 studies were excluded, including 20 case/reports series, five studies with non-SCAPE, and three reviews. Among the five studies included in the analysis of the effect of HDN in treating SCAPE, two studies were prospective cohorts, two were retrospective cohorts, and one was an experimental RCT, with sample sizes ranging from 20 to 67 patients, as revealed in Table 1.

**Table 1:** Characteristics and results of the studies evaluating the effect of HDN in treating SCAPE AKI: acute kidney injury; BP: blood pressure; CI: confidence interval; HDN: high-dose nitroglycerin; HR: hazard ratio; ICU: intensive care unit; IV: intravenous; LDN: low-dose nitroglycerin; MACE: major adverse cardiac events; MV: mechanical ventilation; RCT: randomized controlled trial; SCAPE: sympathetic crashing acute pulmonary edema; μg/minute: micrograms per minute

Author	Year	Study design	Intervention	Outcomes
Mathew et al. [4]	2021	Prospective cohort	25 adult patients received intravenous bolus HDN of ≥600 μg/minute)	96% of the patients normally discharged and 4% was intubated.
Siddiqua et al. [5]	2023	RCT	26 patients received HDN (≥600 μg/minute), 26 LDN (<100 μg/minute)	Patients receiving HDN (>100 mcg/minute) had shorter hospital stay (P < 0.001), and lowered rate of MACE (P = 0.02). MV was significantly lower in HDN comparing to LDN (HR = 0.04; 95% CI: 0.01-0.19; P < 0.05).
Marndi et al. [6]	2024	Prospective cohort	20 patients received HDN (≥600 μg/minute)	Symptoms of all patients resolved within the first six hours of treatment.
Houseman et al. [7]	2023	Retrospective cohort	67 patients received HDN infusion (≥100 μg/minute)	Rates of ICU admission, intubation, acute kidney injury (AKI) at 48 hours, and hypotension were 37%, 21%, 13%, and 4% respectively. MV was significantly lower in HDN comparing to LDN (HR = 0.21; 95% CI: 0.13-0.32; P < 0.05).
Kelly et al. [8]	2023	Retrospective cohort	27 patients received LDN (<100 μg/minute) and 14 received HDN (≥100 μg/minute)	The HDN group reached their blood pressure faster (HR = 3.5, 95% CI: 1.2-10.1). 57% of HDN patients reached their BP target within the first hour of treatment, compared 22% in LDN group (P < 0.05). MV was significantly lower in HDN comparing to LDN (HR = 0.14; 95% CI: 0.04-0.40; P < 0.05).

Mathew et al. presented a prospective cohort study of 25 adults treated with intravenous bolus HDN (≥600 μg/minute). The mean bolus dose of nitroglycerin given was 872 μg, and the mean cumulative dose was 35 mg. No cases of hypotension occurred following the administration of the nitroglycerin bolus dose. Notably, 96% of these patients were discharged without complications, and only 4% required intubation, suggesting a high rate of clinical recovery [4].

Siddiqua et al. performed an RCT comparing 26 patients on HDN (600-1000 mcg bolus followed by starting infusion rate of 100 mcg/minute) to 26 on LDN no bolus, and a starting infusion rate of 20-40 mcg/minute). At 6 hours, symptom resolution was observed in 17 patients (65.4%) in the HDN group, compared to only three patients (11.5%) in the LDN group (P < 0.001). By 12 hours, clinical resolution had occurred in 88.5% of patients receiving the HDN, whereas just 19.5% of those in the LDN group showed improvement. The HDN group experienced a significantly shorter hospital stay (P < 0.001) and a reduced rate of MACE (P = 0.02), highlighting the potential for improved outcomes with higher dosing [5].

Marndi et al. reported on 20 patients in a prospective cohort who all received HDN (≥600 μg/minute). The average initial bolus administered to all patients was 836 µg. All patients experienced symptom resolution within the first six hours of treatment, indicating rapid clinical improvement [6].

Houseman et al. conducted a retrospective cohort study involving 67 patients who received HDN infusions at doses ≥100 μg/minute. Intravenous nitroglycerin was started at a median dose of 100 mcg/minute, with an interquartile range (IQR) of 100 to 200 mcg/minute. The median peak infusion rate during the first hour was 200 mcg/minute (IQR 127.5 to 200 mcg/minute), and the highest recorded dose overall reached 400 mcg/minute. Additionally, 73% of patients were treated with non-invasive positive pressure ventilation (NIPPV), and 48% received either sublingual or intravenous bolus nitroglycerin prior to the continuous infusion of nitroglycerin. The outcomes showed that ICU admission occurred in 37% of patients, intubation in 21%, acute kidney injury (AKI) at 48 hours in 13%, and hypotension in only 4%. These rates suggest that while complications are not negligible, the incidence of severe hypotension remains low with HDN [7].

Kelly et al. retrospectively compared 27 patients in the LDN group (<100 μg/minute) and 14 in the HDN group (≥100 μg/minute). The HDN group achieved target blood pressure more rapidly (hazard ratio (HR) 3.5, 95% CI: 1.2-10.1), with 57% reaching their goal within the first hour versus 22% in the LDN group (P < 0.05) [8].

Discussion

The collective evidence from these studies indicates that HDN is both effective and relatively safe for the acute management of SCAPE. Consistently reported outcomes include more rapid symptom resolution, expedited attainment of hemodynamic goals, reduced incidence of adverse cardiac events, shorter hospital stays, and a decreased need for intubation and MV when compared to lower-dose regimens.

Rapid Symptom Resolution and Discharge Rates

The rapid vasodilatory effect of HDN reduces both preload and afterload, improving cardiac output and pulmonary congestion more efficiently than lower doses. This mechanism underlies the observed rapid clinical improvement. Mathew et al. and Marndi et al. demonstrated that HDN leads to rapid symptom resolution and high rates of uncomplicated discharge [4,6]. In Marndi et al., all patients resolved symptoms within six hours [6], while Mathew et al. reported a 96% normal discharge rate [4]. Similarly, Siddiqua et al. reported that, at six hours, symptom resolution was observed in 17 patients (65.4%) in the HDN group, compared to only three patients (11.5%) in the LDN group (P < 0.001) [5]. This supports the hypothesis that aggressive afterload reduction with HDN can quickly reverse the pathophysiological cascade of acute pulmonary edema.

Faster Achievement of Blood Pressure Targets

Prompt blood pressure control is critical in SCAPE to prevent further cardiac decompensation. HDN’s potent vasodilatory action accelerates hemodynamic stabilization, explaining the faster achievement of BP targets. Kelly et al. found that HDN patients were significantly more likely to reach blood pressure targets within the first hour compared to LDN (57% vs. 22%, P < 0.05). The HR (HR = 3.5) further quantifies this benefit [8].

Shorter Hospitalization and Lower MACE

By rapidly reversing pulmonary edema and stabilizing patients, HDN likely reduces the duration of critical illness and subsequent complications, leading to shorter hospitalizations and fewer adverse events. Siddiqua et al. showed that HDN not only shortens hospital stay but also reduces the incidence of major adverse cardiac events compared to LDN [5].

Low Rates of Hypotension and AKI

While hypotension is a theoretical risk of high-dose vasodilators, careful monitoring and titration in the acute setting appear to mitigate this risk, as reflected in the low incidence of adverse events. Houseman et al. reported low rates of hypotension (4%) and AKI (13%) at 48 hours, suggesting that HDN is generally well-tolerated when carefully titrated [7].

*Need for Mechanical Ventilation* 

A key finding emerging from multiple studies is the association between HDN administration and improved patient outcomes, particularly in terms of MV. The HR for MV was notably lower in the HDN group, suggesting that higher doses may confer protective effects against respiratory deterioration. These findings are corroborated by Siddiqua et al. (HR = 0.04; 95% CI: 0.01-0.19; P < 0.05) [5], Houseman et al. (HR = 0.21; 95% CI: 0.13-0.32; P < 0.05) [7] and Kelly et al. (HR = 0.14; 95% CI: 0.04-0.40; P < 0.05) [8], whose retrospective analyses revealed similar trends: HDN was associated with a lower incidence of MV.

Limitations

Several limitations must be acknowledged. First, the sample sizes in most studies were modest, limiting the statistical power and generalizability of the findings. Only one study was an RCT; the rest were cohort studies, some retrospective in nature, which introduces potential biases such as selection bias and confounding. Additionally, the definition of HDN varied between studies, complicating direct comparisons and synthesis.

Furthermore, the studies did not uniformly report on long-term outcomes, focusing instead on short-term endpoints such as ICU admission, symptom resolution, and hospital discharge. There was also limited reporting on adverse effects beyond hypotension and AKI, and the follow-up periods were generally short.

Finally, the studies were conducted in different clinical settings and may have included patients with varying severity of illness and comorbidities, which could influence outcomes.

## Conclusions

In summary, the reviewed evidence supports the use of HDN as an effective and generally safe intervention for SCAPE, with benefits including rapid symptom relief and improved short-term outcomes. However, larger, multicenter RCTs with standardized dosing protocols and longer follow-up are needed to confirm these findings and further define the risk-benefit profile of HDN in this critical care context.
